# PI3K-mTOR-S6K Signaling Mediates Neuronal Viability via Collapsin Response Mediator Protein-2 Expression

**DOI:** 10.3389/fnmol.2017.00288

**Published:** 2017-09-15

**Authors:** Eun J. Na, Hye Yeon Nam, Jiyoung Park, Myung Ah Chung, Hyun Ae Woo, Hwa-Jung Kim

**Affiliations:** College of Pharmacy, Research Institute of Pharmaceutical Sciences, Ewha Womans University Seoul, South Korea

**Keywords:** mTOR pathways, PI3K, CRMP-2, neuronal outgrowth, pentylentetrazole

## Abstract

Collapsin response mediator protein (CRMP)-2 and the mammalian target of rapamycin complex 1 (mTORC1) signaling pathway are associated with common physiological functions such as neuronal polarity, axonal outgrowth and synaptic strength, as well as various brain disorders including epilepsy. But, their regulatory and functional links are unclear. Alterations in CRMP-2 expression that lead to its functional changes are implicated in brain disorders such as epilepsy. Here, we investigate whether changes in CRMP-2 expression, possibly regulated by mTOR-related signaling, correlates with neuronal growth and viability. Inhibition of mTOR and/or phosphoinositol-3-kinase (PI3K) led to deceased p-S6K, and p-S6 signals also reduced CRMP-2 expression. These changes corresponded to inhibition of neuronal viability and proliferation in cultured hippocampal HT-22 cells under both basal serum-free and serum- or insulin-induced mTOR pathway-activated conditions. CRMP-2 expression tended to be increased by mTOR activation, indicated by an increase in p-S6/S6 level, in pentylentetrazole (PTZ)-induced epileptic rat hippocampal tissues was also significantly reduced by mTOR inhibition. Knockdown of CRMP-2 by si-RNA reduced the neuronal viability without changes in mTOR signaling, and overexpression of CRMP-2 recovered the glutamate-induced neurotoxicity and decrease of mTOR signaling in HT-22 cells. In conclusion, CRMP-2 protein expression controlled by the PI3K-mTOR-S6K signaling axis exerts its important functional roles in neuronal growth and survival.

## Introduction

Collapsin response mediator protein-2 (CRMP-2) is widely expressed in the central nervous system (CNS), and its critical functional involvement in axonal neurite promotion and neuronal polarity through direct or indirect interaction with cytoskeleton molecules such as tubulins has long been demonstrated (Fukada et al., 2000; Charrier et al., 2003). More recent studies have expanded the roles of CRMP-2 to neuronal migration and differentiation, axonal transport, neurotransmitter release, and neuronal survival, through interaction with its various binding partners such as the voltage-gated calcium channel Cav2.2 (Brittain et al., 2009), N-methyl-D-aspartate (NMDA) receptor and Na^+^/Ca^2+^ exchanger (Brustovetsky et al., 2014). The phosphorylation status of CRMP-2, regulated by various kinases, modulates its functional roles (Yamashita et al., 2012) such that the signaling cascades of p-Akt/p-GSK3β for the decrease in p-CRMP-2 mediate synaptic formation and functional axonogenesis in the CNS (Fang et al., 2014). Therefore, alterations in the expression level and phosphorylation state of CRMP-2, which lead to its functional changes, are likely related to various neurological diseases including Alzheimer’s disease (AD; Wang et al., 2013; Hensley and Kursula, 2016), ischemia (Chung et al., 2005), epilepsy (Wilson et al., 2014), schizophrenia (Liu et al., 2014) and alcohol-drinking behaviors (Liu et al., 2017).

Mammalian target of rapamycin (mTOR), a serine-threonine protein kinase of the phosphatidylinositol 3-kinase (PI3K)-related kinase family (Wullschleger et al., 2006), is a master regulator that integrating energy, nutrients, and other multiple upstream signals to regulate numerous important physiological functions including cell growth, survival, homeostasis, and tissue regeneration and repair (Dazert and Hall, 2011; Zoncu et al., 2011; Laplante and Sabatini, 2012). There are two mTOR complexes (mTORCs): mTORC1 and mTORC2. Both are associated with a number of diseases those including neurodegeneration (Dazert and Hall, 2011; Zoncu et al., 2011; Laplante and Sabatini, 2012). Various ligands such as growth factors and neurotransmitters activate target membrane receptors, and transduce the activated signal, resulting in the modulation of a pathway involving PI3K and Akt, which also plays vital roles in cell growth, proliferation, neuroplasticity, and survival (Read and Gorman, 2009). There exist multiple upstream signals of mTOR including insulin signaling through PI3K and AKT. The PI3K-phosphoinositide-dependent protein kinase (PDK1)-Akt and other signals phosphorylate and inactivate tuberous sclerosis complex 1/2, leading to the activation of Rheb by serving as a GTPase exchange factor and consequently the activation of mTORC1 (Manning and Cantley, 2003). Downstream signals of mTOR include p70S6 kinase 1 (S6K) and eukaryotic initiation factor 4E-binding protein 1 (4EBP), which are phosphorylated and activated by mTORC1. Phosphorylation of S6K, particularly at threonine 389, has been widely used to indicate mTORC1 activity. S6K and 4EBP regulate translational initiation and control protein synthesis. Activation of S6K by mTOR is followed by phosphorylation of a downstream ribosomal protein, S6, at serine 235/236, an alternative marker for mTOR activity, to promote protein translation (Fingar et al., 2004; Hay and Sonenberg, 2004).

In the CNS, beneficial or detrimental roles of upstream and downstream signaling of mTOR pathways in synaptic activity or neuronal survival according to physiological and pathological conditions remain debatable (Takei and Nawa, 2014; Bockaert and Marin, 2015). mTOR signaling activation is involved in neuronal synaptic repair following traumatic brain injury (Chen et al., 2007), in the process of memory and learning via protein synthesis-dependent strengthening of synapses (Ma and Blenis, 2009; Maiese et al., 2012), and in the prevention of Aβ-induced synaptic plasticity impairment (Ma et al., 2010) and inflammatory cell death (Shang et al., 2012). Inhibition of mTOR by rapamycin to induces autophagic neuronal death and impair synaptic plasticity in cultured neurons and hippocampal slices in models of AD (Ma et al., 2010). Hyperactivation of mTOR is associated with several types of epilepsy (Citraro et al., 2016). Chronic hippocampal infusion of rapamycin reduces neuronal cell death and mossy fiber sprouting in a rat model of temporal lobe epilepsy. Inhibition of mTOR activity can limit aggressive behavior as well as seizure activity, indicating that epilepsy may be closely linked mTOR signaling (Wong, 2009; Zeng et al., 2009; Huang et al., 2010; McMahon et al., 2014).

Although both CRMP-2 and the mTOR signaling pathways are associated with common physiological and pathological conditions, the link between their functional roles has been examined in only a few studies. Morita and Sobue (2009) reported that S6K is a downstream effector for mTOR-controlled axon formation, and the mTOR-S6K pathway tightly controls the translational expression of several neuronal polarity proteins including CRMP-2. The mTOR pathways may also affect the phosphorylation state of CRMP-2, resulting in its functional change, since mTOR activity can be regulated by the PI3K-Akt-GSK-3β pathway, which is known to regulate CRMP-2 phosphorylation status in neuronal degeneration (Wakatsuki et al., 2011; Xiong et al., 2012; Fang et al., 2014).

In the present study, we investigate whether the change in expression or phosphorylation of CRMP-2 is controlled via mTOR-related signaling pathways that correlate with relevant functions. Our results demonstrate that, in cultured hippocampal neuronal cells and epileptic rat hippocampal tissues, the expression rather than the phosphorylation of CRMP-2 is increased, and thus, tightly regulated through the downstream signaling axis of PI3K-mTOR-S6K. This leads to the functional enhancement of neuronal viability.

## Materials and Methods

### Chemicals, CRMP-2 siRNA and cDNA and Reagents

Rapamycin, insulin, LY294002, 3-methyladenine (3-MA), and pentylentetrazole (PTZ) were purchased from Sigma-Aldrich (St. Louis, MO, USA). Oligonucleotides for control siRNA (SN-1002) and three CRMP-2 siRNAs (#1, SN-1351187: 5′ CAC CAU UUA CUC CUG AUG U 3′; #2, SMARTpool^®^ J-041965-12: 5′ GGG AAU GAC AUC CGC UGA U 3′; #3, SN-1351189: 5′ CUA AUA GCA AGA CCA GUU A 3′) were synthesized by Bioneer Corporation (Daejeon, South Korea) or purchased from Thermo Fisher Scientific (SMARTpool^®^, Waltham, MA, USA). The V5-tagged full length CRMP-2 cDNA that was originally provided by professor S. Strittmatter (Department of Neurology, School of Medicine, Yale University, New Haven, CT, USA) was inserted into pcDNA3.1(+). The CRMP-2-pcDNA3.1 was transformed in *E.Coli* XL10 Blue super competent cells and spread on LB agar plates containing 100 μg/ml ampicillin for selection. After 15 h of incubation at 37°C, positive colonies were cultured in LB media and the grown colonies were purified by QIAGEN plasmid miniprep kit (QIAGEN, Hilden, Germany).

### Neuronal Cell Culture and Transfection

HT-22 immortalized mouse hippocampal cells were received from Prof. Inhee-Mook (Seoul National University, South Korea). HT-22 cells were cultured and maintained in Dulbecco’s modified Eagle’s medium supplemented with 10% fetal bovine serum, 100 units/ml penicillin, and 100 μg/ml streptomycin at 37°C in humidified conditions under 5% CO_2_. The medium was changed twice weekly, and cultures were split in the ratio of 1:10 weekly. For CRMP-2 siRNAs and CRMP-2-pcDNA3.1 transfection experiments, the cells were seeded at 1 × 10^5^ cells/well in 6-well plates, cultured for 24 h, and transfected with each siRNA (30 nM) or each plasmid (50 ng) using Dharma-FECT transfection reagent (Thermo Fisher Scientific, Waltham, MA, USA) or Lipofectamine™ 2000 transfection reagent (Life Technologies Co., CA, USA) for 24 h according to the manufacturer’s instructions. Transmitted light microscopy observation of cells treated with agents for 24 or 48 h in serum-free or serum-containing medium was performed using an EVOS^®^ microscope (Thermo Fisher Scientific Inc., Waltham, MA, USA) before sample preparation. All images are presented in magnification of 200× (Achromat Oil Objectives 20×; Numerical Aperture 0.40; Working Distance: 6.80 mm). CRMP-2 siRNA oligonucleotides (si-CRMP-2: 5′ CAC CAU UUA CUC CUG AUG U 3′) and control-siRNA were selected based on published synthetic siRNA sequences by the manufacturer (Bioneer Corporation, Daejeon, South Korea).

### Determination of Neuronal Number and Viability

HT-22 cells treated with agents were washed with phosphate buffered saline (PBS), harvested and mixed with a 0.4% trypan blue solution, and viable cells in the cell suspension were counted using a hemocytometer. Cell viability was assessed using a cell counting kit-8 (CCK-8; Dojindo Laboratories, Kumamoto, Japan). Cells were plated in 96-well plates at a density of 8 × 10^3^ cells/well, incubated for 24 h, and subsequently treated with various concentrations of agents for 24 or 48 h. The solution was removed from each well and replaced with 2-(2-methoxy-4-nitrophenyl)-3-(4-nitrophenyl)-5-(2,4-disulfophenyl)-2H-tetrazolium, monosodium salt (WST-8), was added to each well and incubated at 37°C for 1 h. The absorbance wavelength was then measured at 450 nm using a VERSAmax tunable microplate reader (Molecular Devices, Sunnyvale, CA, USA).

### Animals and Surgery

Sprague-Dawley Male rats (230–240 g body weight) were purchased from Orient Bio Department (Kyungki-do, South Korea). The animals were housed individually in a temperature- (20 ± 1°C) and relative humidity-controlled environment, maintained on a 12 h light/12 h dark cycle. All animal experiments were conducted according to ethical procedures and approved by the Institutional Animal Care and Use Committee of Ewha Women’s University (Approval No. Ewha-IACUC 2013-01-041).

Rats were anesthetized with zoletil (20 mg/kg) and xylazine (9.5 mg/kg) and placed in a stereotaxic apparatus. A Hamilton syringe attached to a Nanomite Injector Syringe Pump (Harvard Apparatus, Holliston, MA, USA) was used to inject the rats with rapamycin (2, 5, or 10 nM, 4 μl) or vehicle (saline, 4 μl) intracerebroventricularly at coordinates of 0.75 mm posterior, ±1.2 mm lateral, and −3.5 mm ventral, relative to the bregma. 24 h following surgery, PTZ (75 mg/kg) was intraperitoneally administered, 6 h later, rats were sacrificed and the hippocampal tissues were quickly excised and extracted for Western blotting analysis.

### Sample Preparation and Western Blotting

Cells were lysed by modified RIPA buffer (pH 8.0, 50 mM Tris-HCl, 150 mM NaCl, 1% NP-40, 0.25% deoxycholate, 1 mM EGTA, and 1% protease inhibitor cocktail immediately prior to use). The whole-cell lysate was prepared by centrifugation at 12,200× *g* for 20 min at 4°C, and the supernatant was collected. Hippocampal tissues were collected in a cold lysis buffer (1% Triton X-100, 1 mM EDTA in PBS and protease inhibitor cocktail), homogenized and put on ice for 20 min, and then centrifuged at 10,000× *g* for 10 min at 4°C. Protein content was determined using a BCATM protein assay kit (Thermo Fisher Scientific), and assessed by Western blotting analyses. Equal aliquots of the samples were denatured at 100°C, separated by 8–10% sodium dodecyl sulfated-polyacrylamide gel electrophoresis, and blotted to polyvinylidene fluoride membranes (Millipore Corporation, Billerica, MA, USA). Membranes were incubated in a blocking buffer containing 5% bovine serum albumin in Tween-containing Tris-buffered saline for 1 h at room temperature. Immunodetection was performed by incubating membrane blots overnight at 4°C separately with 1:1000 dilution of the following primary antibodies: anti-CRMP-2 (IBL, Gunma, TS, Japan), anti-phospho mTOR (p-mTOR, Ser2448, Cat. #2971), anti-phospho p70S6 kinase (p-S6K, Thr389, Cat. #9209), anti-phospho S6 (p-S6, Ser235/236, Cat. #4858), anti-mTOR(Cat. #2972), anti-S6K (Cat. #9209), anti-S6 (Cat. #2317), and anti-synapsin-I (Cat. #5297) (All from Cell Signaling, Dallas, TX, USA). For chemiluminescent detection, membrane blots were incubated with 1:2000 of horseradish peroxidase-conjugated secondary antibody for 2 h at room temperature. Data collection and processing of the integrated optical density of the bands were performed with a LAS-3000 luminescent image analyzer and IMAGE GAUSE software (Fujifilm, Tokyo, Japan).

### Statistical Analysis

The significance of the differences in all data among the three groups was evaluated with either one-way analysis of variance (ANOVA) or Kruskal-Wallis test, followed by *post hoc* multiple comparisons with Tukey test. The *in vivo* pentylenetetrazole data were additionally evaluated with two-way ANOVA. All statistical tests were performed with GraphPad Prism version 5.0d (GraphPad Software, Inc., La Jolla, CA, USA). A two-tailed *p*-value of <0.05 was considered statistically significant. All results are expressed as the mean ± SEM from at least three independent experiments.

## Results

### mTOR Pathway-Dependent Regulation of CRMP-2 and Synapsin-I Expression, and Neuronal Viability Under Both Serum-Free and Serum-Activated *In Vitro* Conditions

**Figure 1:**
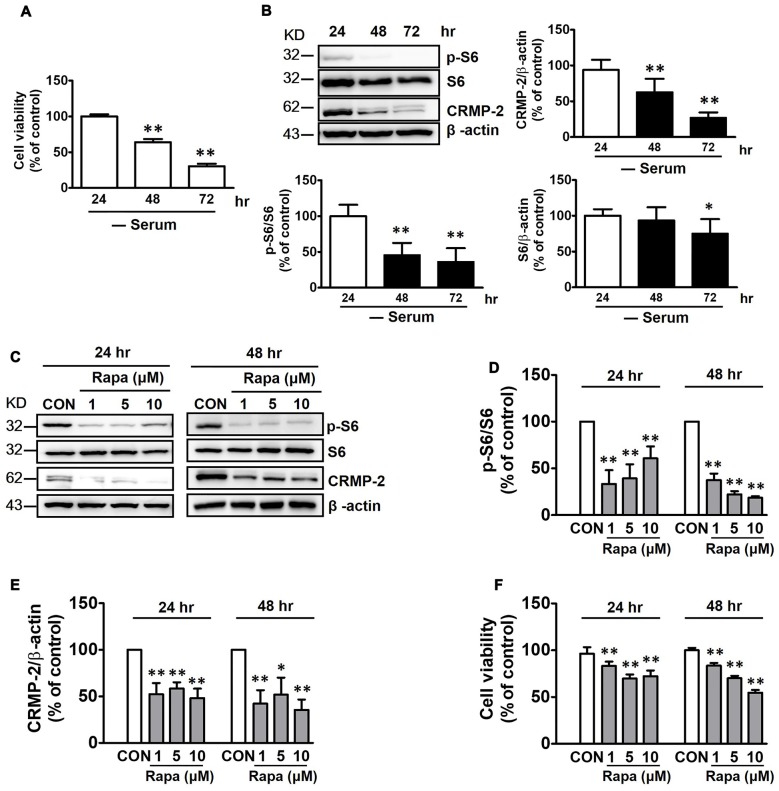
Effects of serum starvation and mammalian target of rapamycin (mTOR) inhibition by rapamycin on mTOR signals, collapsin response mediator protein-2 (CRMP-2) expression, and cell viability in HT-22 cells. Cells were cultured in serum-starved media for 24, 48 and 72 h, and cell viability was compared using a cytotoxicity assay kit (CCK-8) **(A)**, and the p-S6/S6 and CRMP-2 levels were analyzed by Western blotting **(B)**. The cells cultured for 24 h and 48 h in serum-starved media were exposed to rapamycin (1, 5, 10 μM) for the next 24 h, and the p-S6/S6 and CRMP-2 levels were analyzed by Western blotting, and subsequently quantified **(C–E)**, and cell viability was also analyzed **(F)**. The quantitative data are the mean ± SEM of six experiments. Significance values indicate *p < 0.05 and **p < 0.01 vs. 24 h-control **(A)**, and vs. 24 h- or 48 h-vehicle control (CON).

mTOR is not stimulated in the fasting state, and basal mTOR activity might be important in maintaining homeostasis of cellular viability (Wullschleger et al., 2006). HT-22 hippocampal cell viability was reduced by increasing time of exposure to serum-free medium from 24 h to 72 h (Figure 1A). In addition, both CRMP-2 protein expression and mTOR activity were concurrently reduced, as indicated by a significant blocking effect on the basal level of phosphorylated S6 (p-S6), a downstream substrate of S6K and mTOR. Expression of the S6 signal itself was downregulated under long-term serum-deprived condition (Figure 1B). Whether the mTOR pathway controls the basal level of CRMP-2 expression in neuronal cells was further determined by examining the effects of rapamycin, a mTOR inhibitor. Exposure of the neuronal cells to rapamycin (1, 5, or 10 μM) for 24 h and 48 h in serum-free medium resulted in a marked inhibition of basal mTOR activity, as indicated by a significant blocking effect on the basal level of p-S6, producing concurrent reduction in the basal expression level of total CRMP-2 (Figures 1C–E). The basal level of p-CRMP-2 was also decreased (data not shown), likely due to the decreased level of CRMP-2 protein. Significant reduction in neuronal viability was concomitantly seen (Figure 1F). These results imply that mTOR signaling is involved in the control of CRMP-2 protein expression, which affects neuronal viability under energy-depleted conditions.

**Figure 2:**
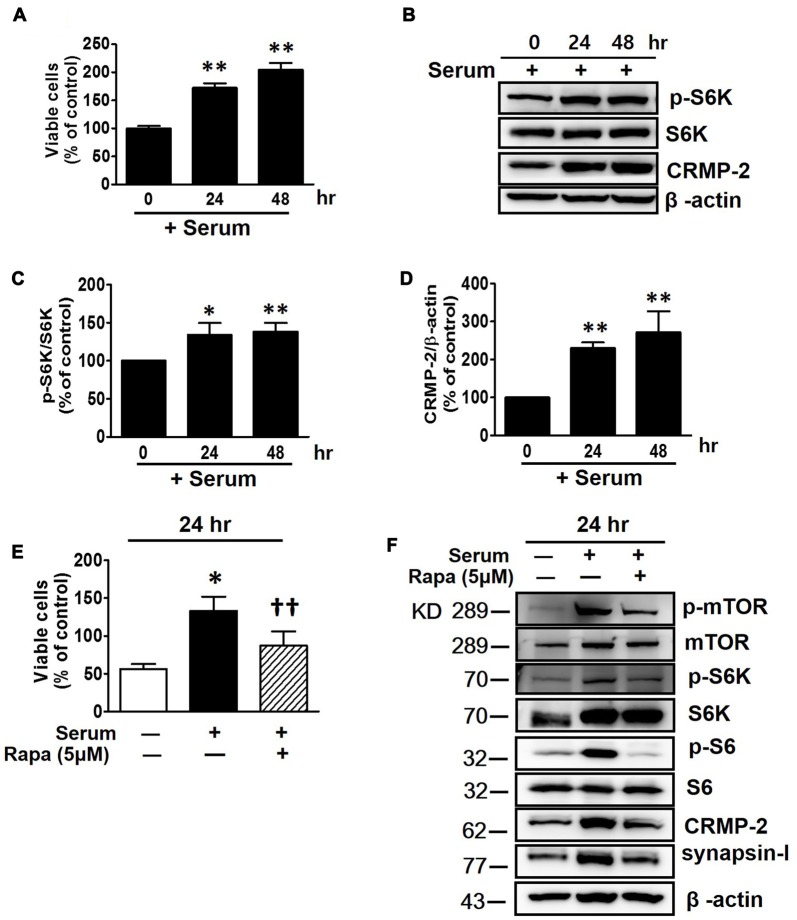
Effects of serum and mTOR inhibition by rapamycin on serum-induced mTOR activity, CRMP-2 and synapsin-I expression and neuronal proliferation in HT-22 cells. Cells were cultured for 24 and 48 h in serum-containing media, and viable cell levels were compared using a CCK-8 **(A)**, and the p-S6K/S6K and CRMP-2 levels were analyzed by Western blotting, and subsequently quantified **(B–D)**. Cells in serum-free media were switched to culture in serum containing media in the absence or presence of rapamycin (5 μM) for 24 h, and viable cell levels were assayed **(E)** and p-mTOR/mTOR, p-S6K/S6K, p-S6/S6, CRMP-2 and synapsin-I were analyzed by Western blotting **(F)**. The quantitative data are the mean ± SEM of five experiments. Significance values indicate *p < 0.05 and **p < 0.01 vs. 0 h + serum control **(A,C,D)** or vs. 24 h − serum control; ^††^*p* < 0.01 vs. 24 h + serum control.

Serum provides nutrients and growth factors needed for activation of mTOR signaling. The viability of HT-22 cells were increased with increased incubation time in serum-containing medium (Figure 2A), in contrast to the decreased viability in serum-free conditions (Figure 1A). As expected, the mTOR signaling (p-S6K) was significantly increased following incubation of the neuronal cells for 24 and 48 h in serum-containing medium, with the significantly enhanced CRMP-2 expression level correlating well with mTOR pathway-activation (Figures 2B–D). Serum-induced increase in neuronal viability was inhibited by mTOR inhibition by rapamycin (Figure 2E). Moreover, when the serum-activated mTOR signaling pathways were blocked by rapamycin, as indicated by changes in the phosphorylation of downstream substrates (p-mTOR, p-S6K and pS6), not only the expression of CRMP-2 but also a synaptic activity marker, synapsin-I, were changed in accordance with the stimulatory and inhibitory states of mTOR downstream signaling pathways (Figure 2F). Although serum exposure undergo β-actin level change to some extent, our quantification data indicate that serum-induced activation of mTOR signaling along with the increases of CRMP-2 and synapsin-I protein levels (Supplementary Figure S1). These results provide evidence for the role of CRMP-2 as a downstream effector controlled by the mTOR signaling pathways, which exert functional influences on neuronal viability.

### Involvement of Insulin-Induced PI3K-mTOR Signaling Activation in the Regulation of CRMP-2 and Synapsin-I Expression, and Neuronal Survival

**Figure 3:**
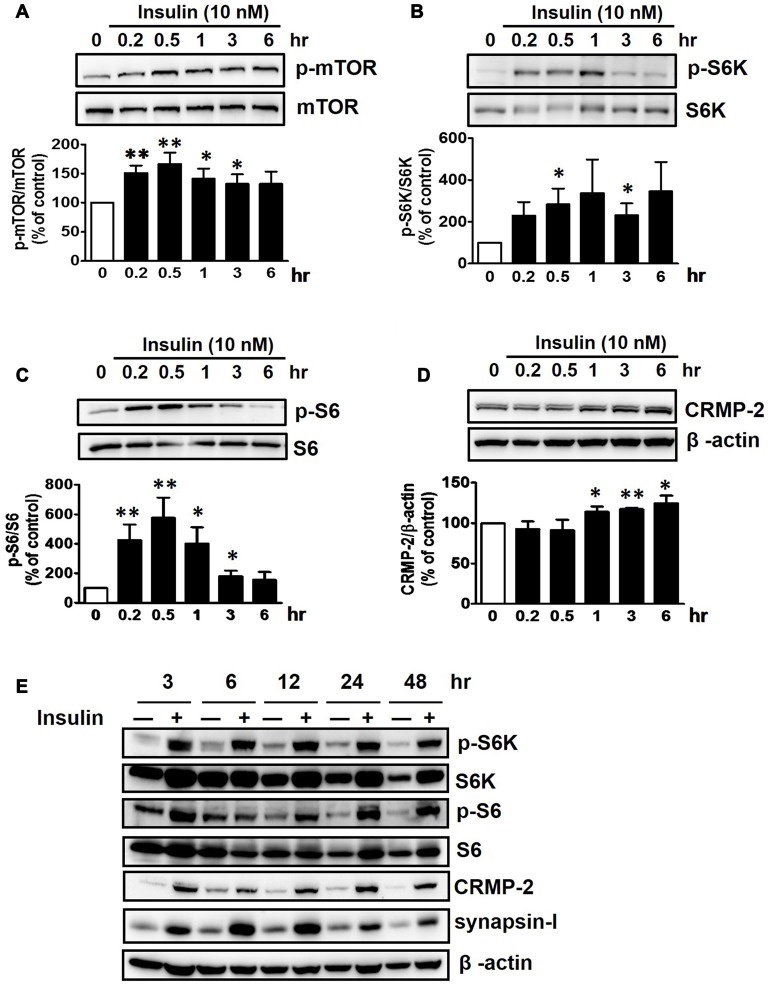
Insulin-induced stimulation of mTOR signals and CRMP-2 expression in HT-22 cells. Cells were treated with insulin (10 nM) for 0.2–48 h in serum-free condition. The p-mTOR/mTOR, p-S6K/S6K, p-S6/S6 and CRMP-2 levels were separately analyzed by Western blotting, and subsequently quantified for 0.2–6 h- treated cells **(A–D)**. In other set of experiments with insulin-treated cells for 3–48 h, the p-mTOR/mTOR, p-S6K/S6K, p-S6/S6, CRMP-2 and synapsin-I levels were simultaneously analyzed by Western blotting **(E)**. Data are the mean ± SEM of six experiments. Significance values indicate *p < 0.05, and **p < 0.01 vs. vehicle control.

**Figure 4:**
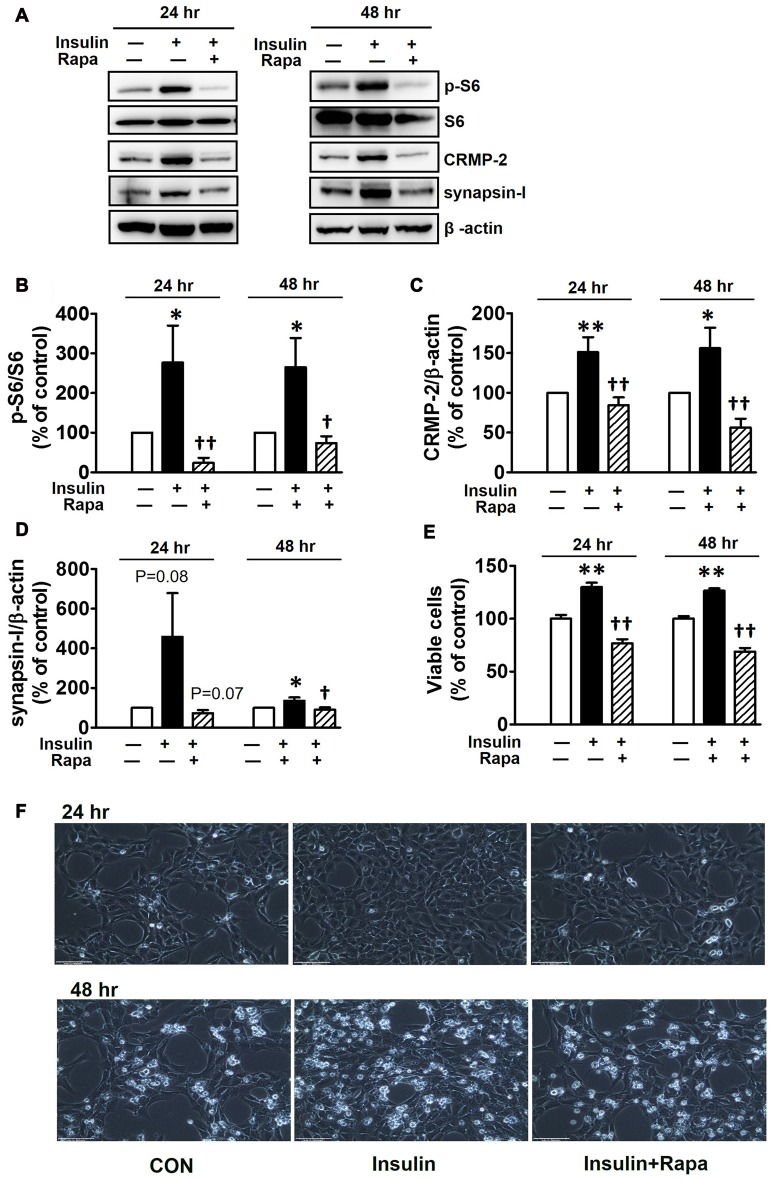
Inhibitory effects of rapamycin on insulin-induced mTOR activity, CRMP-2 and synapsin-I expression and neuronal growth in HT-22 cells. Cells were treated with insulin (10 nM) in the absence and presence of rapamycin (5 μM) for 24 and 48 h. The p-S6K/S6K, p-S6/S6, CRMP-2 and synapsin-I levels were analyzed by Western blotting, and subsequently quantified **(A–E)**. Viable cells were assayed using a CCK-8 **(E)**. Representative data for microscopic observation (200×, Scale bar, 100 μm) of cells are presented **(F)**. The quantitative data are the mean ± SEM of six experiments. Significance values indicate *p < 0.05, and **p < 0.01 vs. vehicle control: ^†^*p* < 0.05 and ^††^*p* < 0.01 vs. insulin control.

Insulin is a well-known upstream regulator of mTOR activity. The insulin signaling pathway connecting to the PI3K-mTOR pathway is essential in the regulation of cell growth, survival and homeostasis under physiological and pathological conditions (Inoki et al., 2012; Dibble and Cantley, 2015). In the CNS, insulin has been shown to promote neuronal outgrowth and survival, the synaptic network, and synaptic plasticity (Nemoto et al., 2011). Control protein synthesis and autophagy through downstream PI3K-Akt-mTOR pathways that are known to be dysregulated in AD and epilepsy (Ma and Blenis, 2009; Ma et al., 2010; Talbot et al., 2012; Nixon, 2013). To investigate whether insulin-activated mTOR signaling also controls CRMP-2 protein expression and functions in neurons, HT-22 cells were treated with insulin (10 nM) for 0.2–48 h. As shown in Figure 3, insulin enhanced mTOR activation, reflected by increases in p-S6K and p-S6, for up to 48 h, although the time patterns for p-S6K and p-S6 signals were somewhat different. The expression levels of CRMP-2 and synapsin-I were also increased by insulin treatment for up to 48 h. Insulin-induced activation of mTOR pathway and concomitant increases in CRMP-2 and synapsin-I expression levels were completely abolished by rapamycin at both the 24 and 48-h (Figures 4A–D). The insulin-enhanced neuronal proliferation/growth and its marked reduction by rapamycin was also observed in the cell viability assay (Figure 4E) and microscopic observations (Figure 4F). These results further suggested that CRMP-2 expression involved in changes in synapsin I expression and neuronal proliferation can be regulated by insulin downstream of the PI3K-Akt-mTOR pathways.

**Figure 5:**
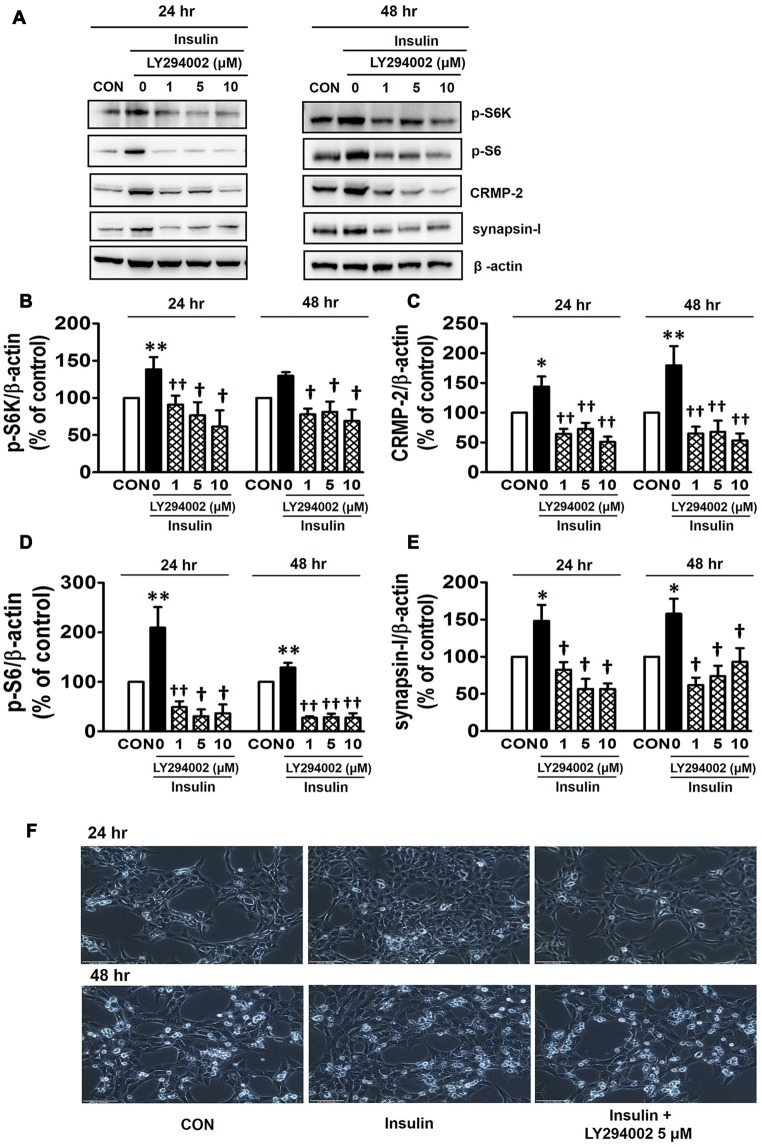
Inhibitory effect of LY294002 on insulin-induced mTOR activity, CRMP-2 and synapsin-I expression, and neuronal growth in HT-22 cells. Cells were treated with insulin (10 nM) in the presence of a phosphoinositol-3-kinase (PI3K) inhibitor, LY294002 (1, 5, 10 μM), for 24 h. The p-S6K, p-S6, CRMP-2 and synapsin-I levels were analyzed by Western blotting, and quantified **(A–E)**. Representative data for microscopic observation (200×, Scale bar, 100 μm) of cells are presented **(F)**. The quantitative data are the mean ± SEM of five experiments. Significance values indicate *p < 0.05, and **p < 0.01 vs. vehicle control: ^†^*p* < 0.05 and ^††^*p* < 0.01 vs. insulin control.

**Figure 6:**
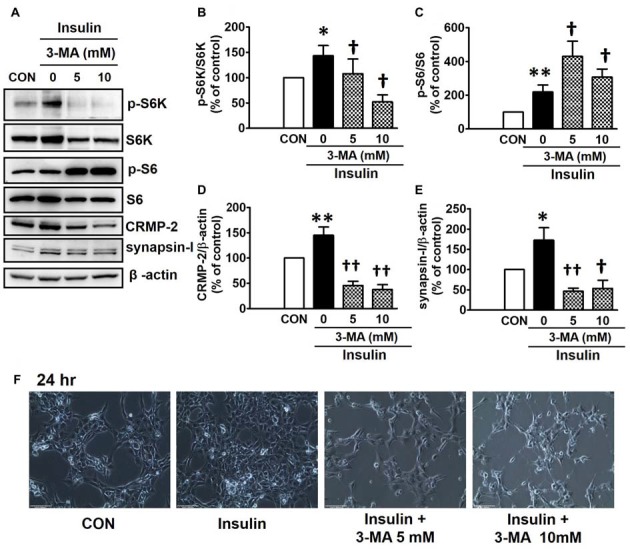
Inhibitory effect of 3-methyladenine (3-MA) on insulin-induced mTOR activity, CRMP-2 and synapsin-I expression and neuronal growth in HT-22 cells. Cells were treated with insulin (10 nM) in the presence of a PI3K inhibitor, 3-MA (5, 10 mM), for 24 h, and the p-S6K/S6K, p-S6/S6, CRMP-2 and synapsin-I levels were analyzed by Western blotting, and quantified **(A–E)**. Representative data for microscopic observation (200×, Scale bar, 100 μm) of cells are presented **(F)**. The quantitative data are the mean ± SEM of six experiments. Significance values indicate *p < 0.05, and **p < 0.01 vs. vehicle control: ^†^*p* < 0.05 and ^††^*p* < 0.01 vs. insulin control.

PI3Ks are signaling molecules that act as upstream regulators of mTOR and, as upstream regulators of autophagy inhibited by mTOR activation (Wu et al., 2009). To examine the involvement of PI3Ks in mTOR activity regulating the CRMP-2 expression, the PI3K inhibitors, LY294002 and 3-MA, were evaluated for their effects on mTOR signaling, and CRMP-2 and synapsin-I expression in HT-22 cells. The pan-PI3K inhibitor LY294002 has been shown to block insulin-induced PI3K-Akt and PI3K-S6K activation differently, which are upstream and downstream events of mTOR I (Adi et al., 2001). 3-MA has been used to inhibit autophagy, which is a downstream event of mTOR (Wu et al., 2009). Insulin (10 nM)-induced mTOR activation indicated by increased levels of both p-S6K and p-S6 was completely blocked by LY294002 at all concentrations (1, 5, 10 μM) and exposure times (24 and 48 h) tested (Figures 5A,B,D). At the same time, the insulin-induced increases in expression levels of CRMP-2 (Figures 5A,C) and synapsin-I (Figures 5A,E), were all significantly reduced by LY294002. The concurrent inhibition of the PI3K-mTOR pathway and the reduction of CRMP-2 expression by LY294002 also correlated well with lowered neuronal proliferation/survival, as indicated by microscopic observations (Figure 5F). Similar to the effect of LY294002, the insulin-induced phosphorylation of S6K, existing immediately downstream of mTOR activation, was completely blocked by another PI3K inhibitor, 3-MA (5, 10 mM; Figures 6A,B). The p-S6 increased by insulin was further enhanced by 3-MA (Figures 6A,C). In additional experiment, HT22 cells were exposed to 3-MA (5 mM), LY (1 μM), 3-MA (5 mM) + LY (1 μM) or 3-MA (5 mM) + rapamycin (5 μM) for 24 h to confirm the 3-MA results. Again 3-MA inhibited mTOR signals (p-S6K), except p-S6, and 3-MA in combination with LY or rapamycin inhibited all the signals (Supplementary Figure S2). This may reflect multiple effects of 3-MA besides PI3K inhibition (Caro et al., 1988; Xue et al., 2002), and the fact that S6 phosphorylation is controlled not only by S6K but also by other signaling pathways such as mitogen-activated protein kinase (Roux et al., 2007). CRMP-2 and synapsin-I protein levels increased by insulin were completely blocked by 3-MA (Figures 6A,D,E). In particular, the CRMP-2 and synapsin-I expression levels reduced by 3-MA were significantly lower than the control levels.

### Involvement of mTOR Signaling in the Regulation of CRMP-2 Expression *In Vivo* in Normal and Pentylentetrazole-Induced Epileptic Rat Hippocampal Tissues

**Figure 7:**
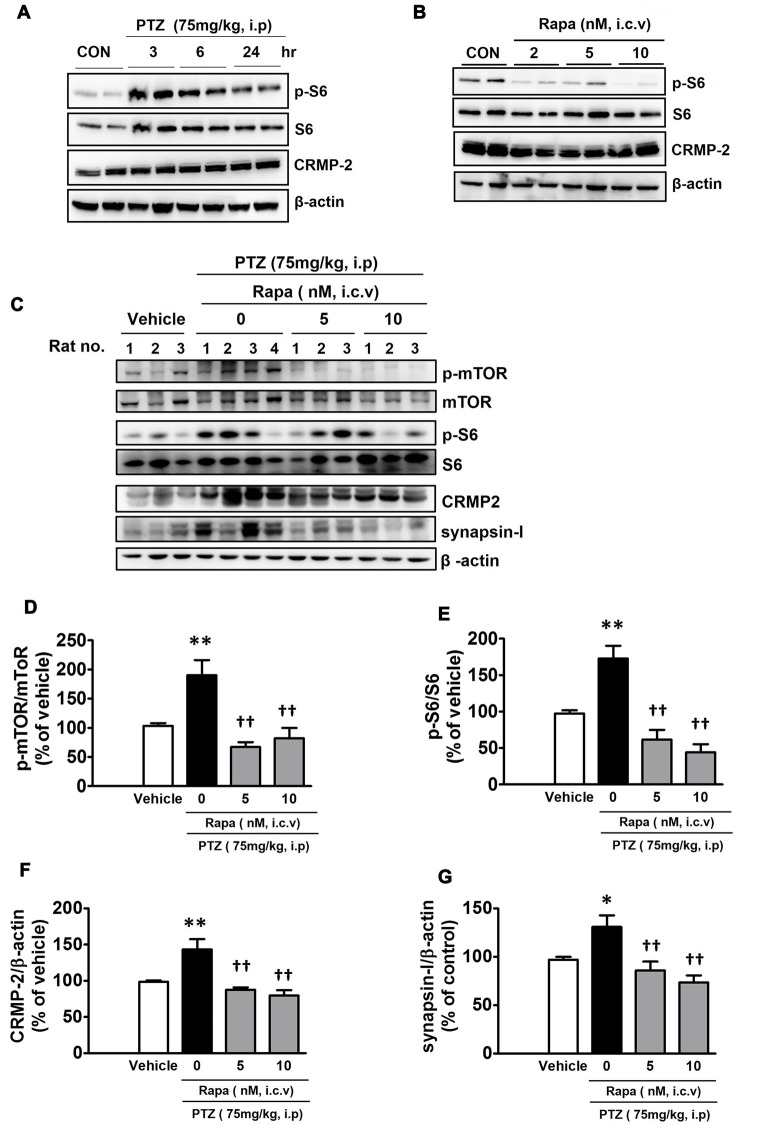
Effect of pentylenetetrazole and mTOR inhibition by rapamycin on mTOR signals, and CRMP-2 and synapsin-I expression in rat hippocampal tissues. Pentylentetrazole (PTZ, 75 mg/kg) was intraperitoneally administered to rats, which produced acute seizures, and hippocampal tissues were extracted at indicated times following PTZ treatment **(A)**. Rats were treated with intraventricular injection of rapamycin (2, 5, 10 nM) and 24 h later the hippocampal tissue was extracted **(B)**, and the p-S6/S6 and CRMP-2 levels were analyzed by Western blotting **(A,B)**. Rats were pretreated with intraventricular injection of rapamycin (5, 10 nM) 24 h prior to PTZ administration, and 6 h thereafter the hippocampal tissue was extracted and analyzed by Western blotting and subsequently quantified for mTOR/mTOR, p-S6/S6, CRMP-2, and synapsin-I **(C–G)**. The quantitative data are the mean of ± SEM of all data from 9 to 10 rats from seven independent experiments (1–7th sets), evaluated with one-way analysis of variance (ANOVA). Significance values indicate *p < 0.05, and **p < 0.01 vs. vehicle control; ^††^*p* < 0.01 vs. PTZ control. Data were additionally evaluated with two-way ANOVA (1–5th sets vs. 6–7th sets; data not shown).

The mTOR signaling pathway has significant and distinct impacts on neurological diseases including epilepsy (Wong, 2013). Intraperitoneal injection of PTZ (75 mg/kg), which has been shown to cause acute epileptic behavioral seizures, activated the mTOR pathway, as reflected by an increased signal of p-S6. The p-S6 was the highest at 3 h and gradually lowered, however, it still remained elevated until 24 h after PTZ injection (Figure 7A). This observation indicates that the duration of PTZ-induced mTOR activation greatly outlasted the period of behavioral seizure activities observed following PTZ injection. Penetylenetetrazole, an antagonist at gamma-aminobutyric acid receptors (GABA_A_), induces acute seizures, however, it does not lead to significant pathological changes or spontaneous epilepsy following a single injection in rodents (Wong et al., 2003). Rats were profiled for 2 h following each PTZ treatment and characterized by stage: stage 0, no change in behavior; stage 1, chewing; stage 2, gazing and head nodding; stage 3, unilateral forelimb clonus, scratching, and twitching; stage 4, rearing with bilateral forelimb clonus; stage 5, widespread muscle spasms, rearing with bilateral forelimb clonus, and falling backwards; stage 6, death (Racine et al., 1972). PTZ-injected rats achieved stage 4 of generalized tonic-clonic seizures during the monitoring periods (data not shown). To confirm the role of mTOR signaling in the modulation of CRMP-2 expression *in vivo*, rats were treated with rapamycin (2, 5, or 10 nM, i.c.v) 24 h prior to injection of vehicle or PTZ, and hippocampal tissues were extracted 6 h after the PTZ injection. Rapamycin produced distinct reductions in the basal p-S6 signal and the CRMP-2 expression level in control (vehicle-treated) rat hippocampal tissues (Figure 7B). The PTZ-induced increase in mTOR pathway signal (p-S6/S6) was also significantly inhibited by rapamycin (Figures 7C–E). In addition, rapamycin pretreatment significantly reduced the levels of CRMP-2 and synapsin-I proteins enhanced in the PTZ-treated rat hippocampal tissues (Figures 7F,G). These findings demonstrate that mTOR pathways could also control CRMP-2 expression and synaptic activity in PTZ-induced epileptic rat brain tissues, similar to that observed in cultured neuronal cells, implicating the potentially important role of CRMP-2 regulated by mTOR in epilepsy, although significant changes by rapamycin in the PTZ-induced behavioral seizure activity were not observed in this study.

### Effects of CRMP-2 Silencing by siRNA and CRMP-2 cDNA Overexpression on Neuronal Viability and the mTOR Signaling Pathway

**Figure 8:**
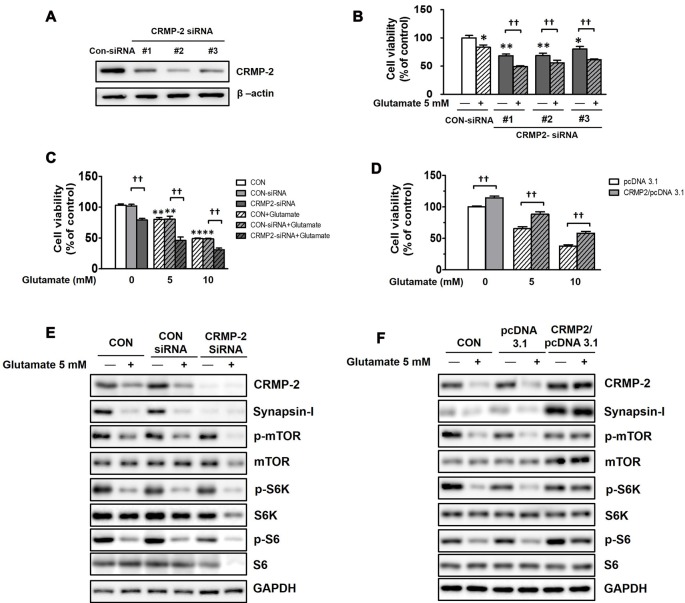
Effect of CRMP-2-siRNA, CRMP-2-cDNA and glutamate on mTOR activity, neuronal viability, synapsin-I and CRMP-2 expression, and mTOR signals in HT-22 cells. Cells were transfected for 6 h with control-siRNA, CRMP-2-siRNAs (#1–3; 30 nM), control pcDNA3.1 or CRMP-2-pcDNA3.1 (50 ng). Post 24 h of transfection, the un-transfected (CON), control- and CRMP-2 siRNA (#1–3 in **A,B** and #1 in **C,E**)- or cDNA **(D,F)**-transfected cells were exposed to glutamate (Glu, 5 or 10 mM) for 24 h. Cell viability was analyzed using a CCK-8 assay **(B–D)**, and the CRMP-2, synapsin-I, p-mTOR/mTOR, p-S6K/S6K, p-S6/S6 levels were analyzed by Western blotting **(E,F)**. Viability data are the mean ± SEM of 5–8 independent experiments (each triplicate). Significance values indicate *p < 0.05, **p < 0.01 vs. un-transfected (CON), CON-siRNA control (-Glu) or pcDNA3.1 control (-Glu): ^††^*p* < 0.01 vs. CRMP-2-siRNA (-Glu) or CRMP-2-pcDNA3.1 (-Glu).

To examine whether CRMP-2 protein expression itself directly plays a critical role in neuronal survival/death and synaptic activity, and whether it is regulated by mTOR-dependent pathways, CRMP-2 depletion was performed using siRNA methodology. HT-22 hippocampal cell viability was significantly decreased (by 20–32%) following CRMP-2 silencing by transfection with three different CRMP-2-siRNA oligonucleotides (30 nM) for 24 h (Figures 8A,B). Glutamate is excitotoxic to various neuronal types including HT-22 cells by contributing to intracellular Ca^2+^ homeostasis and reactive oxygen species formation (Tan et al., 1998). Exposure of un-transfected (CON), control-siRNA- and empty pcDNA3.1 vector-transfected HT-22 cells to glutamate (5, 10 mM) for 24 h produced significant dose-dependent reduction of the viability (Figures 8C,D). The glutamate-induced decrease in neuronal viability was significantly exacerbated by approximately 30–40% further in CRMP-2-silenced cells (Figures 8B,C). Concurrently with the glutamate induced decreased in neuronal viability, exposure of both control- and CRMP-2 siRNA-transfected cells to glutamate decreased expressions of the CRMP-2, synapsin-I, p-mTOR, p-S6K, p-S6 (Figure 8E). The neurotoxic effect of CRMP-2 depletion alone by CRMP-2-siRNA transfection for 24 h without glutamate was similar to the extent induced by glutamate (5 mM) exposure for 24 h to the control-siRNA-transfected cells and accompanied by the dramatic disappearance of synapsin-I expression (Figure 8E), strongly implying the critical role of CRMP-2 in the synaptic protein level. The mTOR signals were not changed by CRMP-2 depletion alone (Figure 8E), suggesting that CRMP-2 is not an upstream regulator of mTOR signaling, but instead a downstream effector controlled by mTOR signaling pathways. Contrary to the CRMP-2-siRNA effect, overexpression of CRMP-2 by transfection of CRMP-2-pcDNA3.1 increased the viability and synapsin-I expression, compared to those in the empty pcDNA3.1 vector-transfected cells. Furthermore, CRMP-2 overexpression also significantly rescued neuronal viability (Figure 8D), and recovered levels of synapsin-I and mTOR signals (p-mTOR, p-S6K, p-S6; Figure 8F) in the glutamate-induced neurotoxic condition.

## Discussion

This study presents the link between CRMP-2 and the mTOR signaling pathway, both of which are commonly associated with various physiological and pathological conditions. The findings indicate that the mTOR-S6K and PI3K signaling pathways control CRMP-2 expression, which plays important roles in neuronal growth/survival and synaptic activity in both *in vitro* and *in vivo* systems.

mTORC1-regulation of CRMP-2 expression, neuronal viability, and synaptic protein level was indicated by the rapamycin-induced inhibition of mTORC1 downstream signaling (S6K and S6) that was accompanied by concomitant downregulated expression of CRMP-2 and synapsin-I, which occurred simultaneously to a reduction in neuronal viability in serum-starved and serum- or insulin-activated conditions in HT-22 hippocampal cells (Figures 1–4). We did not focus on the possibility that CRMP-2 phosphorylation is controlled via mTORC1 downstream signaling pathways, due to the substantially decreasing effect on total CRMP-2 protein levels by mTOR inhibition (Supplementary Figure S3).

Serum deprivation itself inhibits mTOR and activate proteolysis, whereas long-term mTOR inhibition or serum deprivation, or sustained inactivation of insulin-like growth factor 1 signaling, may also trigger secondary responses and reduce autophagy (Renna et al., 2013). In our serum-starved (up to 72 h) neuronal culture conditions, however, mTORC1 downstream signaling was decreased, as indicated by reduced p-S6 signals, and was accompanied by a decrease in CRMP-2 protein levels as well as reduced neuronal viability (Figures 1A,B), although whether serum deprivation alone stimulates CRMP-protein degradation or inhibits protein translation was not pursued in this study. During serum deprivation for 24 and 48 h, further inhibition of mTORC1 by rapamycin also correlated well with further decreases in both CRMP-2 protein levels and neuronal viability (Figures 1C–F).

The close correlations among mTORC1 signaling, expression levels of CRMP-2, and neuronal growth were further confirmed in the presence of serum and insulin which activate upstream and downstream mTORC1 signaling pathways. Activation of mTORC1 downstream signaling (p-mTOR, p-S6K, and p-S6) by serum and insulin induced corresponding increases in the expression levels of synapsin-I and CRMP-2, proteins commonly representing synaptic strength, and that was accompanied by increased neuronal growth and viability in HT-22 hippocampal cells. According to a report by Morita and Sobue (2009), activation of mTOR induces local translation of some polarity-related genes including CRMP-2 in primary cultured neurons. Therefore, our data showing enhanced expression levels of CRMP-2 and synapsin-I by serum or insulin treatment could result from the translational regulation of these proteins promoted by downstream signals of mTORC1 activation. Insulin prevents cell death in rat retina neuronal cells due to serum deprivation by activating of mTOR and S6K (Punzo et al., 2009); we observed that neuronal cell number enhancement in insulin-treated HT-22 cells in serum-free medium was blocked by mTOR inhibition, which was accompanied by changes in p-mTOR, p-S6K, and p-S6 (Figure 4). Functional roles of mTOR upstream and downstream pathways in neuronal survival and synaptic activity depend on various physiological and pathological conditions. Induction of PI3K-AKT-mTORC1 signaling promotes survival and blocks excessive autophagy that leads to cell death. On the other hand, inhibition of mTORC1 may stimulate autophagy of damaged or toxic proteins and the promote cell survival via feedback activation of mTORC2 on AKT (Sarbassov et al., 2005; Benjamin et al., 2011; Gan et al., 2011; Oh and Jacinto, 2011). Rapamycin is generally selectively sensitive to mTORC1, but not to mTORC2, however, prolonged rapamycin treatment (100 nM, 24 h also inhibits mTORC2 assembly (Sarbassov et al., 2006) and insulin-induced mTORC2 activation (Copp et al., 2009)). Whether both mTORC1 and mTORC2 were inhibited under our experimental conditions of treatment with rapamycin (5–10 nM) for 24 and 48 h is uncertain, however, reduced neuronal survival/growth by rapamycin treatment was consistently observed throughout our study.

There are many reports demonstrating that insulin promotes gene expression that leads to the synthesis of various proteins, and the insulin downstream pathway, class I PI3K-mTORC1-S6K, is thought to play essential roles in the expression of proteins that modulate cell death such as apoptotic cascades and/or promoting the cell survival. Therefore, we examined whether the expression of CRMP-2 and synapsin-I, and accompanying changes in neuronal viability can also be regulated by PI3K as an upstream activator of mTOR signaling. Our observation that insulin-induced mTOR activation (measured by the p-S6K to S6K ratio) was completely abolished by the addition of PI3K inhibitors, LY294002 (Figure 5) and 3-MA (Figure 6) implies the existence of a connection between insulin signaling and the PI3K-mTORC1 pathway in HT-22 cells.

Class I and III PI3Ks have been characterized as major PI3Ks that control mTORC1 activity and autophagy in different manners (Yu et al., 2015). The general paradigm is that stimulation of class I PI3K-Akt through membrane receptors such as the insulin receptor, activates mTORC1-S6K and thereafter, inhibiting autophagy (Wu et al., 2009). The activity of class III PI3K, also known as human vacuolar protein sorting 34 (Vps34), is essential in triggering autophagy via the control of autophagosome formation and maturation (Petiot et al., 2000; Backer, 2008). However, exceptions to this paradigm have also been reported. The catalytic subunit of class I PI3K was shown to act as a positive regulator of autophagy (Dou et al., 2010), and class III PI3K/Vps34 was shown to activate S6K1 via nutrients such as amino-acids, through the activation of mTORC1, which suppress autophagy (Byfield et al., 2005; Nobukuni et al., 2007; Wu et al., 2009). A pan inhibitor of PI3K, LY294002 that blocks both class I and III PI3Ks, suppressed the insulin-stimulated mTOR-dependent S6K-S6 signaling and produced a corresponding inhibition of the enhancement of insulin-induced CRMP-2 and synapsin-I expression (Figure 5). 3-MA also almost completely blocked insulin-stimulated S6K phosphorylation, indicating its inhibitory effect on class I PI3K-mTOR signaling, similar to the effect of LY294002, and concomitantly reduced CRMP-2 and synapsin-I levels and neuronal growth (Figure 6). Although 3-MA has been widely used to suppress autophagy due to its ability to inhibit autophagosome formation by interfering with class III PI3K (Petiot et al., 2000) and suppressing protein degradation and cell migration (Ito et al., 2007), 3-MA has also been demonstrated to inhibit Akt as a result of class I PI3K interference, which leads to the suppression of the mTOR pathway (Lin et al., 2012). Our LY294002 and 3-MA data strongly indicate that insulin-induced CRMP-2 and synapsin-I expression can be regulated through PI3Ks-S6K, upstream and downstream of the mTORC1 signaling pathway. The reason for the further enhancement of insulin-induced p-S6 levels by 3-MA (Figure 6E) is unclear, although 3-MA blocked the insulin-induced increases in p-S6K and expression of CRMP-2 and synapsin-I. Wu et al. (2009) reported that 3-MA blocks class I PI3K persistently, whereas its suppressive effect on class III PI3K is transient. Whether the result of the 3-MA-induced increase in p-S6 is a phenomenon affected by feedback modulations following transient inhibition of the class III PI3Ks and autophagy was not uncovered. Other possible speculations include the complex overlapping and reverse roles of classes I and III PI3Ks in TORC1 activity and protein synthesis or degradation, and effects of 3-MA on multiple cellular events besides PI3K inhibition (Caro et al., 1988; Xue et al., 2002), and S6 phosphorylation controlled not only by S6K, but also by other signals such as mitogen-activated protein kinase (Roux et al., 2007).

Changes in the TSC1/2 gene responsible for tuberous sclerosis ultimately results in mTOR activity, leading to epilepsy (Cho, 2011; Russo et al., 2012; Chong et al., 2013). In animal models of kainite- or pilocarpine-induced temporal lope epilepsy, phosphorylation of S6, indicating mTOR activation, was reported in cortical and hippocampal tissues (Buckmaster et al., 2009; Zeng et al., 2009; Huang et al., 2010). In the present study, we also demonstrated significant activation of mTOR signaling (p-mTOR and p-S6) in *in vivo* PTZ-induced epileptic rat hippocampal tissues, and the mTOR activation outlasted the seizure period up to 24 h following administration of PTZ. Our data do not exactly correspond to the report by Zhang and Wong (Zhang and Wong, 2012), in which mTOR activity (p-S6) remained elevated for 3–6 h and returned to basal level 16 h after seizure onset by PTZ. Rapamycin injection (10 nM, i.c.v.) 24 h prior to administration of PTZ produced a significant reduction in CRMP-2 expression, accompanied by the marked inhibition of PTZ-induced activation of mTOR signaling, indicated by decreased p-mTOR and p-S6 levels, in hippocampal tissues (Figure 7). It has been reported that mTOR inhibition suppresses the development of various types of epileptogenic changes, indicating the involvement of mTOR signaling in epileptogenesis (Citraro et al., 2016). Our findings demonstrate that mTOR pathways also control CRMP-2 and synaptic protein expression in *in vivo* epileptic rat brain tissues, as seen in cultured neuronal cells, and therefore imply the potentially important role of CRMP-2 regulated by mTOR in epilepsy. A very recent *in vivo* study reported a link between CRMP-2 and mTOR signaling; excessive alcohol consumption increased the translation of CRMP-2 as a target of mTORC1 in the rat nucleus accumbens (Liu et al., 2017).

CRMP-2 may contribute to the pathophysiology of epilepsy and may be a potential therapeutic target for the prevention of epileptogenesis, although its mechanism remains unclear (Quach et al., 2015). It was demonstrated that lacosamide administration attenuates axon sprouting and excitatory synaptic connectivity to deep-layer pyramidal neurons by inhibiting CRMP-2-mediated neuronal outgrowth and axonal sprouting (Wilson et al., 2012). Moreover, an increase in CRMP-2 expression following pilocarpine-induced seizures led to axonal growth and guidance in the formation of mossy fiber sprouting (Lee et al., 2012), implying the crucial role of CRMP-2 in cell proliferation and synaptic activity related to epilepsy.

In experiments with CRMP-2-siRNA-treated HT-22 neuronal cells, we further confirmed that CRMP-2 expression itself could play a crucial role in neuronal viability. No changes of mTOR signals (p-mTOR, p-S6K, p-S6) by CRMP-2 downregulation alone suggest that CRMP-2 is a downstream target rather than an upstream regulator of mTOR signaling pathways. The CRMP-2 function has been speculated to be modified by glutamate receptor-mediated neuronal death (Brustovetsky et al., 2014). Moreover, glutamate-mediated oxidative toxicity has been shown to induce autophagic cell death and inhibit mTOR signals in HT22 cells (Kim et al., 2009; Mao et al., 2016). The extent of the neurotoxic effect induced by glutamate (5 mM) exposure for 24 h in control-siRNA-transfected cells was similar to that induced by CRMP-2-siRNA transfection alone for 24 h. The glutamate-induced decrease in neuronal viability was exacerbated further in CRMP-2-silenced cells (Figure 8C), but rescued in CRMP-2-overespressed cells (Figure 8D). The mTOR signals and CRMP-2 expression levels decreased by exposure to glutamate (Figures 8E,F) was recovered by CRMP-2 cDNA transfection (Figure 8F). Our data strongly demonstrate that CRMP-2 expression is translationally regulated through mTOR downstream signaling pathways, that CRMP-2 play an important role in neuronal viability, and that glutamate inhibits mTOR-S6K-S6 signaling and its downstream event (CRMP-2 translation) to subsequently induce neuronal death.

In conclusion, these results show *in vitro* and *in vivo* evidence that mTOR signaling plays a crucial role in the regulation of CRMP-2 expression, which is involved in neuronal growth/survival.

## Author Contributions

EJN performed experiments with the assistance of HYN, JP and MAC, analyzed the data and wrote the first draft of manuscript. HAW supervised experiments and data analyses. H-JK conceived, designed and supervised the whole research, and wrote the manuscript. All authors reviewed the manuscript.

## Conflict of Interest Statement

The authors declare that the research was conducted in the absence of any commercial or financial relationships that could be construed as a potential conflict of interest.
