# Supplementary material for: PI3K-mTOR-S6K Signaling Mediates Neuronal Viability via Collapsin Response Mediator Protein-2 Expression
Source: Front Mol Neurosci. 2017 Sep 15;10:288. doi: 10.3389/fnmol.2017.00288 (PMC5605571; doi:10.3389/fnmol.2017.00288)
Supplement: Supplementary file 1 [file Data_Sheet_1.docx]

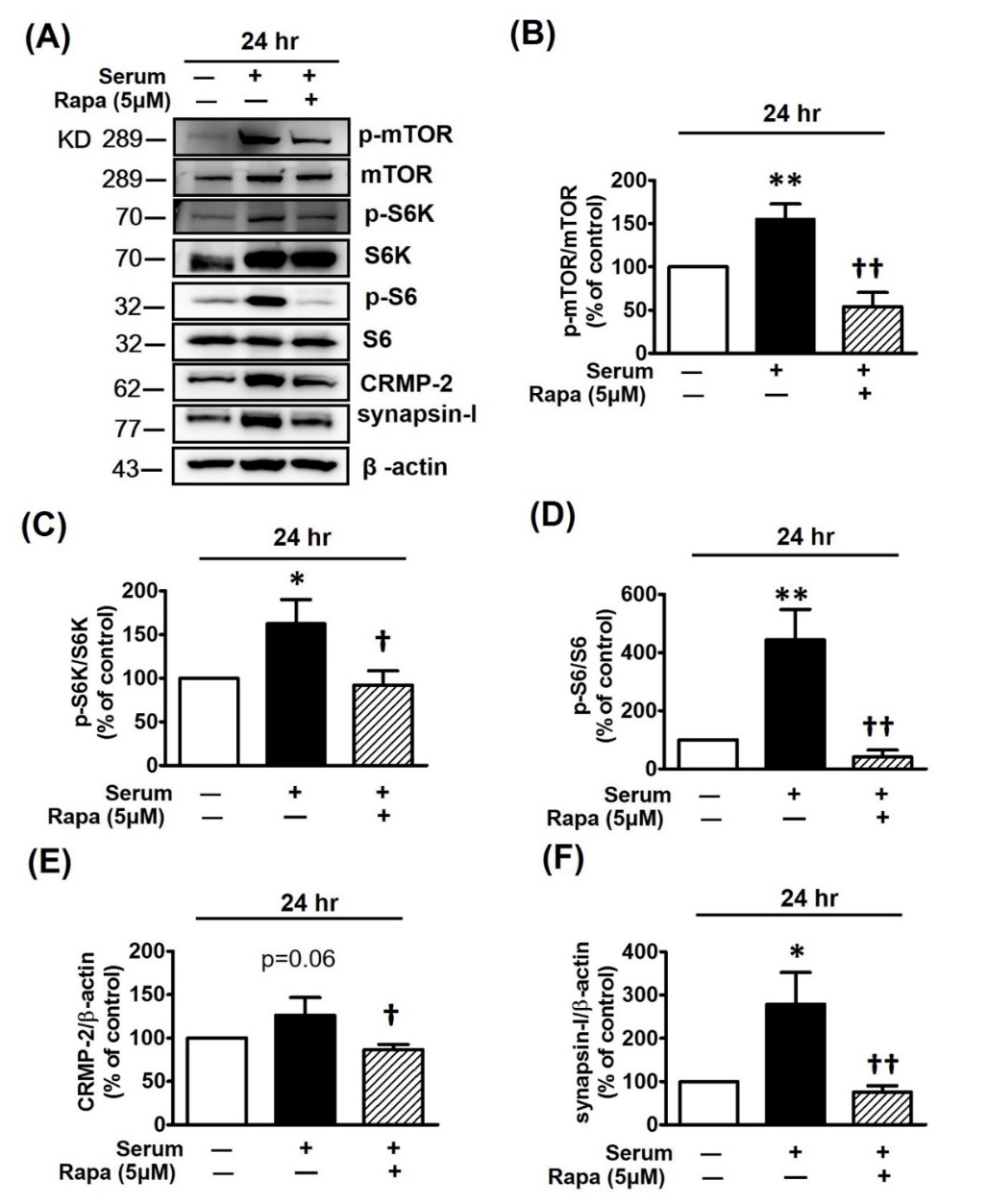


**Supplementary Figure S1.** Effects of mTOR inhibition by rapamycin on serum-induced p-mTOR/mTOR, p-S6K/S6K, CRMP-2 and synapsin-I expression in HT-22 cells. Cells in serum-free media were switched to culture in serum containing media in the absence or presence of rapamycin (5 μM) for 24 hours, and the cell extracts were analyzed by Western blotting (A) and each blot was quantitated (B-F). The quantitative data are the mean ± S.E.M. of 5 experiments. Significance values indicate *p < 0.05 and **p < 0.01 vs. 0 hours + serum control or vs. 24 hours – serum control; †p < 0.05 and ††p < 0.01 vs. 24 hours + serum control.
