# Supplementary material for: PI3K-mTOR-S6K Signaling Mediates Neuronal Viability via Collapsin Response Mediator Protein-2 Expression
Source: Front Mol Neurosci. 2017 Sep 15;10:288. doi: 10.3389/fnmol.2017.00288 (PMC5605571; doi:10.3389/fnmol.2017.00288)
Supplement: Supplementary file 2 [file Data_Sheet_2.docx]

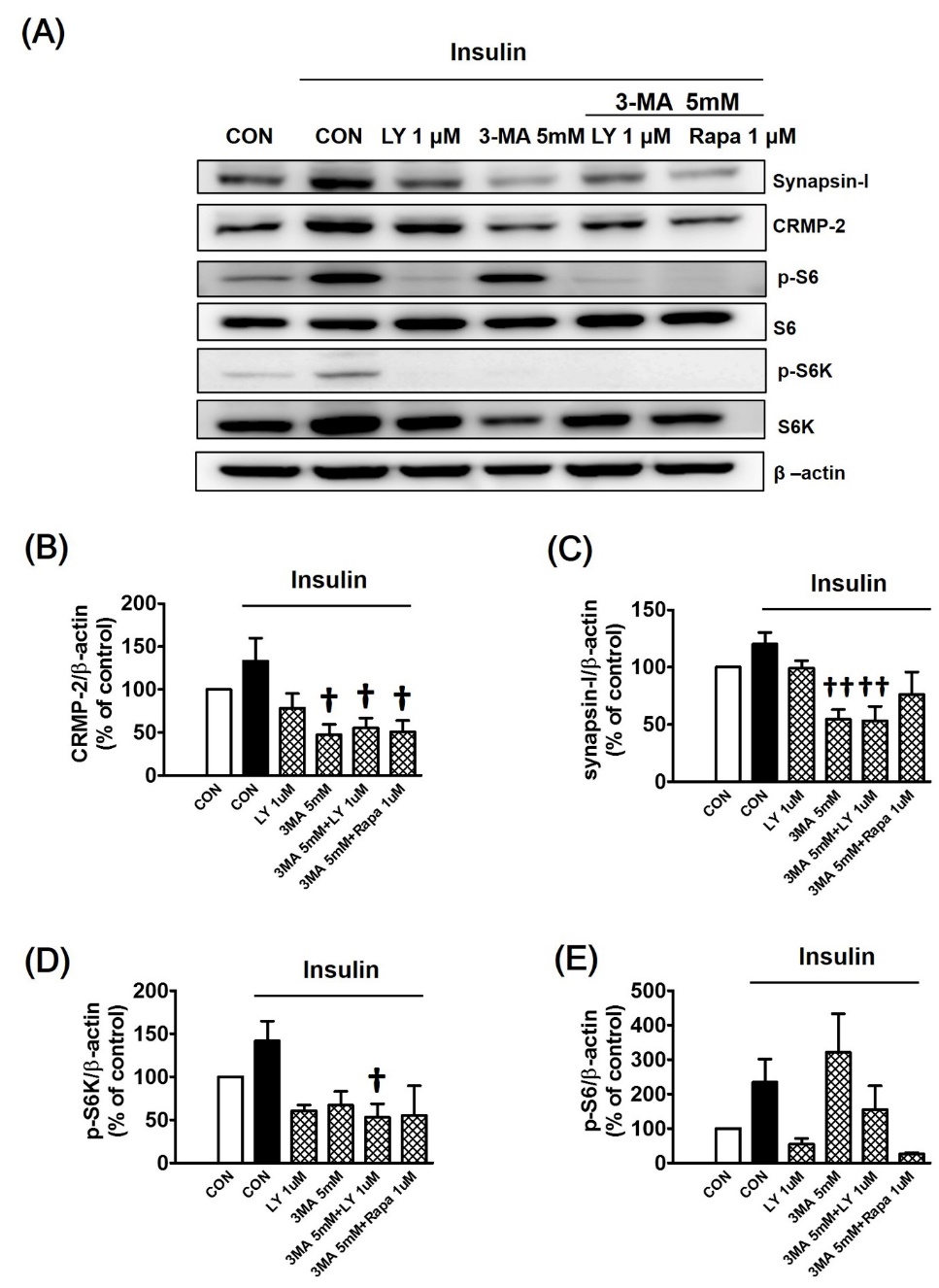


**Supplementary Figure S2. Effect of 3-MA+LY294002 on insulin-induced mTOR activity, CRMP-2 and synapsin-I expression, and neuronal growth in HT-22 cells.** Cells were treated with insulin (10 nM) in the absence and presence of 3-MA (5 mM), LY294002 (1 μM), or 3-MA + LY294002, or 3-MA + rapamycin (5 μM) for 24 hours. The p-S6K, p-S6, CRMP-2, and synapsin-I levels were analyzed by Western blotting (A-E). Significance values indicate †p < 0.05 and †† p < 0.01 vs. insulin control.
