# Supplementary material for: PI3K-mTOR-S6K Signaling Mediates Neuronal Viability via Collapsin Response Mediator Protein-2 Expression
Source: Front Mol Neurosci. 2017 Sep 15;10:288. doi: 10.3389/fnmol.2017.00288 (PMC5605571; doi:10.3389/fnmol.2017.00288)
Supplement: Supplementary file 3 [file Data_Sheet_3.docx]

**
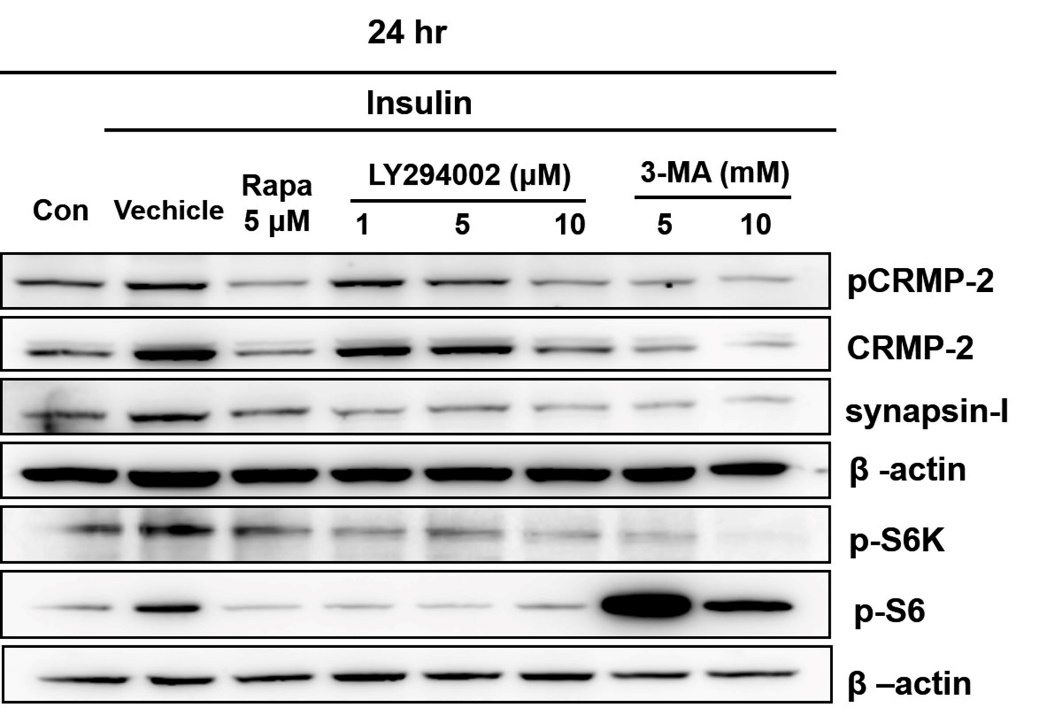
**

**Supplementary Figure S3.** Effects of PI3K or mTOR inhibitors on insulin-induced p-CRMP-2, CRMP-2, synapsin-I, and S6k, and pS6 in HT-22 cells. Cells were treated with insulin (10 nM) in the presence of rapamycin (5 μM), 3-MA (5, 10 mM) or LY294002 (1, 5, 10 μM) and for 24 hours. The p-CRM2, CRMP-2, synapsin-I, p-S6K and p-S6 levels were analyzed by Western blotting.
